# Development of Carbon Black Coating on TPU Elastic Powder for Selective Laser Sintering

**DOI:** 10.3390/ma17133363

**Published:** 2024-07-08

**Authors:** Yu-Deh Chao, Shu-Cheng Liu, Dong-Quan Yeh, Ajeet Kumar, Jung-Ting Tsai, Mayur Jiyalal Prajapati, Jeng-Ywan Jeng

**Affiliations:** 1 High Speed 3D Printing Research Center, National Taiwan University of Science and Technology, No. 43, Sec. 4, Keelung Rd, Taipei 106, Taiwan; hjkhjkhjk86@yahoo.com.tw (Y.-D.C.); d11003003@mail.ntust.edu.tw (S.-C.L.); yehowen70753@gmail.com (D.-Q.Y.); mayurprajapati969@gmail.com (M.J.P.); 2 Department of Mechanical Engineering, National Taiwan University of Science and Technology, No. 43, Sec. 4, Keelung Rd., Taipei 106, Taiwan; tsaij@mail.ntust.edu.tw; 3 Design for Additive Manufacturing & Innovation (DAMI) Lab, Department of Design, Indian Institute of Technology Guwahati, Guwahati 781039, Assam, India; ajeetkumar@iitg.ac.in; 4 Academy of Innovative Semiconductor and Sustainable Manufacturing, National Cheng Kung University, No. 1, University Rd., Tainan 701, Taiwan; 5 Department of Design, Indian Institute of Technology Guwahati, Guwahati 781039, Assam, India; 6 Visiting Scholar, The Extreme Light Infrastructure, ERIC, 252 41 Prague, Czech Republic

**Keywords:** Thermoplastic Polyurethane (TPU), selective laser sintering, laser reflectivity, carbon black-coated TPU

## Abstract

Increased usage of selective laser sintering (SLS) for the production of end-use functional components has generated a requirement of developing new materials and process improvements to improve the applicability of this technique. This article discusses a novel process wherein carbon black was applied to the surface of TPU powder to reduce the laser reflectivity during the SLS process. The printing was carried out with a preheating temperature of 75 °C, laser energy density of 0.028 J/mm^2^, incorporating a 0.4 wt % addition of carbon black to the TPU powder, and controlling the powder layer thickness at 125 μm. The mixed powder, after printing, shows a reflectivity of 13.81%, accompanied by the highest average density of 1.09 g/cm^3^, hardness of 78 A, tensile strength of 7.9 MPa, and elongation at break was 364.9%. Compared to commercial TPU powder, which lacks the carbon black coating, the reflectance decreased by 1.78%, mechanical properties improved by 33.9%, and there was a notable reduction in the porosity of the sintered product.

## 1. Introduction

Additive manufacturing has revolutionized product design by offering unprecedented flexibility and addressing the limitations of traditional manufacturing processes [1,2]. Consequently, elastic polymer materials have emerged as innovative options, particularly in polymer powder additive manufacturing, predominantly through selective laser sintering (SLS) [3]. The inherent flexibility of additive manufacturing has rendered materials solely focused on high strength inadequate for fulfilling all designers’ requirements. As a result, flexible high-polymer materials have gradually gained prominence in the market, catering to producing products capable of energy absorption or demonstrating flexibility, such as protective gear and shoe components [4,5,6]. While rubber was historically the primary material for elastic applications, its lack of recyclability prompted the adoption of thermoplastic polyurethane (TPU), which can be reshaped after melting in various commercial products [7,8].

The material used in the study is thermoplastic polyurethane (TPU), a type of thermoplastic elastomer (TPE) known for its excellent mechanical performance [9,10,11]. TPU combines the mechanical strength of plastics with the elasticity of rubber and can be shaped using traditional processing methods like extrusion, injection molding, and blow molding. With technology advancing, TPU finds applications in various fields, such as footwear, tubing, films, cable coatings, rollers, various automotive components, and consumer electronic wearables [12,13]. In recent years, many companies have focused on developing custom-made insoles, making the material applicable in additive manufacturing to enhance product customization. The primary raw materials for synthesizing TPU involve polymer polyols, diisocyanates, and chain extenders [14]. These three materials react to form block copolymers with soft and hard segments. The polyol forms the soft segment, providing the material with flexibility and high elasticity. The combination of diisocyanates and chain extenders creates the hard segment, imparting excellent mechanical properties to the material [15,16].

In powder bed fusion technology, polymer powder materials primarily utilize Selective Laser Sintering (SLS), whereas pure powder materials require scanning and sintering by high-power CO_2_ lasers. Adding carbon black or flux agents to the powder materials makes using shorter wavelength laser sources possible and reduces the energy required for sintering [17,18]. When using TPU powder manufactured by Sinterit, it was observed that vibration caused the separation between the powder and carbon particles. Through spectral analysis using a spectrometer, it was found that the test results of reflectance exhibited fluctuations. These fluctuations in reflectance cause uneven energy absorption during the laser sintering process, subsequently affecting the bonding strength of the final product [19,20].

This paper explores mixing carbon black in TPU powder for SLS. The addition of carbon black to TPU improves energy absorption, which enables it to be sintered with a low-powered laser. The carbon black coating has been explored before for polyamide 12 (PA12) powders for powder bed fusion to improve other properties. Hong et al. used a two-step approach to fabricate nano-carbon black/PA12 composites using the SLS process. They found that the high laser energy density or high carbon black content can improve the sinterability degree and mechanical properties of the composite materials [21]. In another study conducted by Xi et al., it was found that coating carbon black powder to the irregular particle size PA 12 powders not only improved the flowability of the powder but significantly improved the mechanical performance and reduced structural defects [22]. Hupfeld et al. used a colloidal surface additivation (CSA) method to prepare carbon-coated PA 12 powder to investigate the influence of carbon on the polymer crystallinity and its effects on mechanical properties [23]. Sommereyns et al. investigated carbon coating by both dry and CSA methods on the powder morphology, thermal, and microstructural characteristics [24]. Some other studies have coated carbon nanotubes (CNTs) and have studied their mechanical, electrical, and piezoresistive properties. CNTs significantly improved the mechanical, electrical, and piezoresistive properties of the additively manufactured parts [19,25,26]. Thiele et al. investigated the additive manufacturing of polymer-metal composite structures where they used carbon-doped TPU powder via laser polymer deposition method [27]. Based on the literature it can be inferred that carbon and its allotropes can be used to improve the absorption of processing energy such as lasers, and moderate additions help improve mechanical properties as well. CNT, Carbon fiber, Graphene, etc. are used to improve electrical and thermal conductivity based on their microstructure. To the best of the author’s knowledge, the effect of carbon-coating on TPU powders and its sinterability is yet to be explored, which is the novelty of this study. Another important highlight of the present article is the use of low-power (5W) laser sources. By varying laser power and printing temperature, this study seeks to determine the process parameters for mixed TPU powder in laser sintering, testing its mechanical properties and printability, and the quality of the printed components.

## 2. Material and Methods

### 2.1. Material Selection

**Figure 1:**
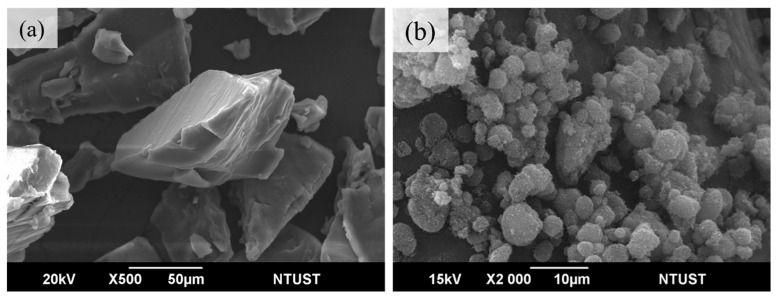
(**a**) TPU powder (**b**) Carbon black.

Figure 1a,b show the SEM images of TPU powders from BASF Elastollan^®^ 1180A and carbon black (Rainbow Pigment Co., Ltd. 75-4849, City of Industry, CA, USA), respectively. The TPU powder has a density of 1.11 g/cm^3^ and carbon black has a density of 1.8 g/cm^3^. Both powders were mixed at different ratios to determine the best SLS printing process.

### 2.2. Experimental Process

This research employed the Sinterit Lisa SLS 3D printer (Kraków, Poland)for additive manufacturing. The material is a mixture of TPU powder and carbon black. The required powder was mixed by heating and stirring, using carbon black to evenly disperse between the powder particles through the active helical blades. Heating facilitated the carbon black coating onto the powder material’s surfaces, achieving uniform adhesion of carbon black onto the TPU powder. This process aimed to improve the separation of the powder materials (TPU and carbon black). After powder preparation, particles were sieved out using a mesh sieve. The selected particle size was prioritized to achieve a good printing condition. Subsequently, tests were conducted by adjusting the printing parameters such as preheating temperature, energy density, and powder layer thickness in the process of carbon black coating on the powder material.

First, the materials are weighed according to experimental proportions. Then, TPU powder is placed into the mixing chamber of the blender (helical blades) and stirred at room temperature for about 10 min using the shear and friction forces of the rotor to disperse the agglomerated powder. Carbon black material is added with time control to ensure the carbon black aggregates are dispersed and fully distributed among the TPU powder. This is followed by heating the material to the melting temperature range of the TPU material and increasing friction and shear forces by momentarily elevating the rotor stirring speed to uniformly coat the carbon black onto the TPU powder surface. The mixed powder is sieved using an LS-300T (Lao Soung Machinery CO., LTD., New Taipei, Taiwan) vibrating sieve with 25 and 120 mesh screens.

Jasco V-670 (JASCO Corporation, Tokyo, Japan) was used to measure the reflectivity of the TPU powder. The instrument Coulter LS 230 from Beckman Coulter, Inc., Brea, CA, USA estimated the particle size distribution (PSD) by employing laser light to illuminate particles suspended in the dispersing phase. The light source is a 732 nm red solid-state laser, and the analysis involves polarized light scattering intensity difference (polarized light intensity difference scattering PIDS technique). The results are calculated and analyzed using Fraunhofer and Mie theories to convert and determine the powder particle sizes. Thermogravimetric Analysis (TGA) was conducted on the mixed TPU material to determine the phase transitions and evaluate overall polymer properties. The Differential Scanning Calorimeter (DSC) analysis depicts the changes in TPU material during heating and explores the material’s heat absorption during the heating stage.

## 3. Results and Discussion

### 3.1. Material Characterization

**Figure 2:**
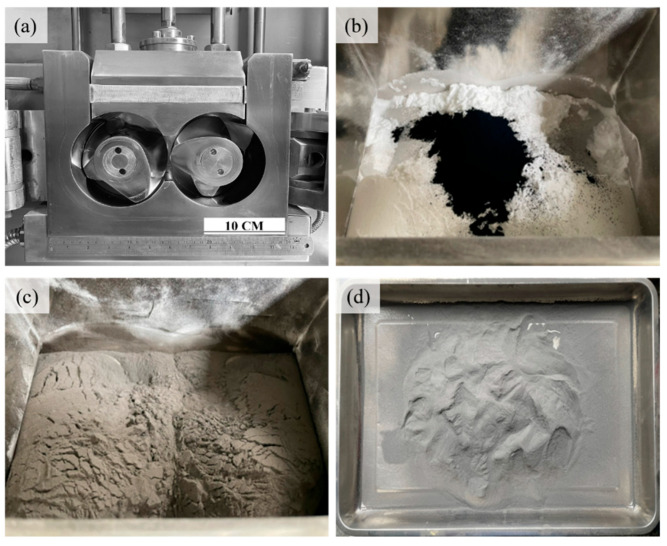
The mixing process of TPU powder and carbon black. (**a**)The mixing chamber (**b**) Adding carbon black in TPU (**c**) Mixed TPU powder with carbon black (**d**) The sieved mixed powder.

Figure 2 shows the preparation process of mixed powder. Figure 2a shows the chamber and size of the mixing chamber. Figure 2b shows the equipment used for mixing carbon black with TPU powder. Figure 2c shows the initial state of adding carbon black with TPU powder. Figure 2d shows that the producing TPU mixed powder coated with carbon black begins with thorough dispersion of carbon black within TPU powder during the initial stages. During the mixing process, temperature control and time are critical during the carbon black coating process. Excessive temperature can initiate a large amount of fusion bonding between powders and generate many kneaded TPU strips, which is undesirable. Subsequent adjustments were made, reducing it to 70 °C, where fusion bonding did not occur. Therefore, the carbon black coating is carried out at this temperature setting. During the sieving process the 25-mesh screen aids in quickly dispersing the agglomerated powder after carbon black coating, while the 120-mesh screen separates large particles, ensuring that the particle size for subsequent printing remains below 125 μm, with a powder yield of 92%.

**Figure 3:**
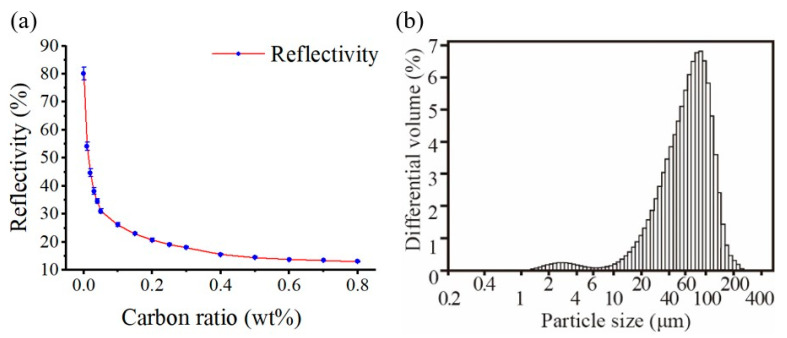
(**a**) Powder reflectivity vs. carbon ratio. (**b**) Powder size distribution.

Figure 3a shows the reflectance values for subsequent laser sintering forming in the 808 nm wavelength range. It was found to be exponentially decreasing as the carbon ratio increased. Lower reflectance values indicate that the material can absorb energy more effectively rather than dissipate it. This, in turn, enhances the feasibility of short-wavelength lasers and increases the likelihood of low-power sintering Table 1. Figure 3b The PSD results are calculated, and the particle size distribution was estimated to be D50, 61.12 µm.

**Figure 4:**
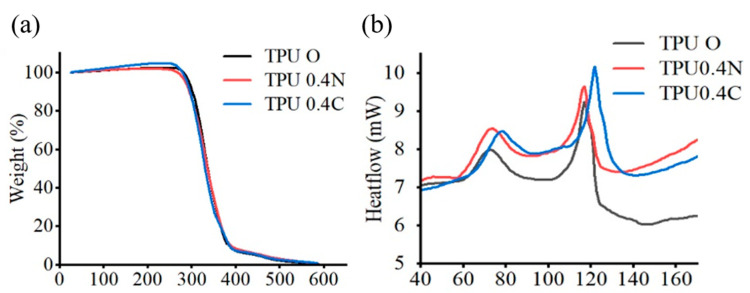
TPU powder for (**a**) TGA and (**b**) DSC results.

Three materials were tested by TGA analysis: TPU O (Virgin powder), TPU 0.4N, and 0.4C (N stands for mixed in room temperature, while C stands for mixed with heating). A heating rate of 20 °C per minute was employed, heating the material from room temperature to 600 °C. Figure 4a shows the TGA analysis. The TGA results indicate that TPU begins to exhibit weight loss around 250 °C, experiences significant weight changes between 300 °C and 400 °C, and approaches zero weight near 600 °C. This information suggests that temperature settings should be kept below 250 °C for subsequent DSC tests to avoid the risk of material degradation.

Figure 4b shows the DSC analysis depicting the changes in TPU material during heating. The DSC test reveals the fundamental heat changes in the material. Based on the TGA test results in Figure 4a, which indicate thermal weight loss in TPU at around 250 °C, the heating temperature for DSC tests was set below 250 °C. The analysis involves heating the material at 10 °C per minute up to 180 °C. The material exhibits exothermal peaks at 72.5 °C and 117.05 °C, corresponding to the crystallized TPU material structure within these temperatures. These findings are crucial for setting the preheating temperature range required for TPU during printing, and help determine the temperature values at which the material undergoes phase transitions and melting.

### 3.2. Printed Dimension in TPU

**Figure 5:**
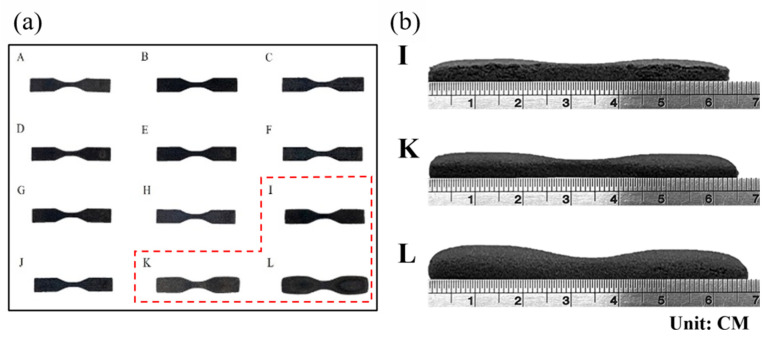
The printed specimens’ diagram. (**a**) Printed specimens’ morphology labeled from A to L. (**b**) Printed specimens of I, L, K side view.

Figure 5a shows that the self-mixed powders were printed with adjusted parameters (A–L), and the weight and density of the samples were measured (Table 2). The dimensions of the nine groups of samples were within standard dimensions with no significant deviations (not including I, K, and L specimens). Figure 5b shows that specimens I, K, and L exhibited significant bulges at the bottom of the samples. The extent of dimensional errors is presented in Table 2. The surfaces of the printed samples were inspected for apparent damage and any abnormalities in appearance dimensions. The subsequent experiments used standard printing parameters to avoid printing errors. The powder coated with carbon black exhibits a uniform carbon coating compared to conventional commercial powder, resulting in lower reflectivity.

### 3.3. Influence of Print Layer Thickness in TPU

**Figure 6:**
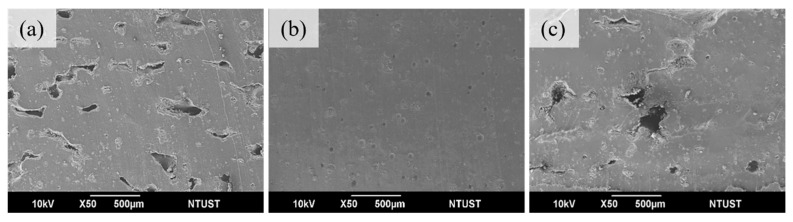
Interlayer adhesion at different layer thicknesses (**a**) 100 μm (**b**) 125 μm (**c**) 150 μm.

The layer thickness selection must be adjusted according to the powder’s particle size distribution. Generally, the layer thickness for laser sintering printing is around 100–150 μm. It is suggested that the layer thickness should be thicker than the average particle size. Changing the layer thickness affected the quality of the final product and the printing time. Therefore, in this experiment, we used these parameters to explore the effect of different layer thicknesses on the printing quality of TPU04C carbon black-coated mixed TPU powder. From Table 3, it can be seen that a layer thickness setting of 125 μm exhibits better tensile strength. Figure 6a shows more pores in the sample cross-section, leading to lower tensile strength. Figure 6b shows that the bonding between layers is good, with only minor pores remaining. Figure 6c shows that due to the larger layer thickness, it is evident that the laser power cannot wholly sinter the powder. Hence, 125 μm was the subsequent experiments’ layer thickness setting parameter.

### 3.4. Influence of Print Temperature and Energy

**Figure 7:**
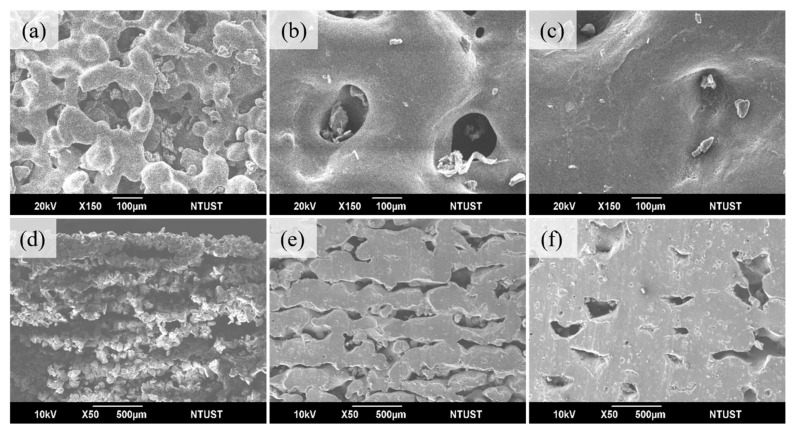
Preheat temperature 35 °C and laser energy (J/mm^2^) used (**a**) 0.011, (**b**) 0.028, (**c**) 0.044 surface; (**d**) 0.011, (**e**) 0.028, (**f**) 0.044 cross-section.

**Figure 8:**
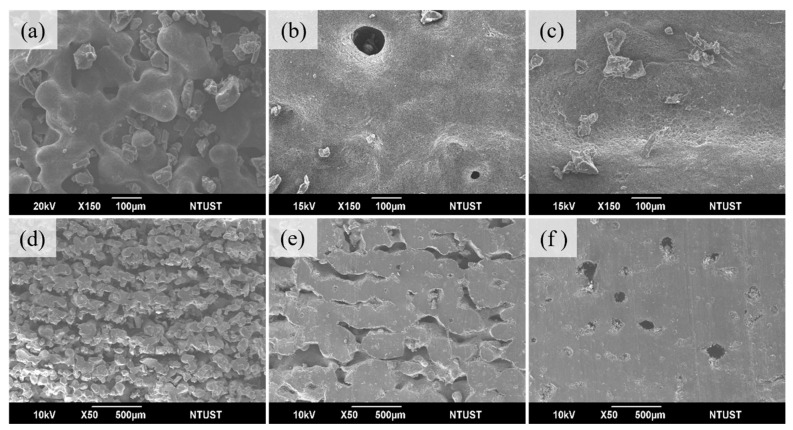
Preheat temperature 55 °C and laser energy (J/mm^2^) used (**a**) 0.011, (**b**) 0.028, (**c**) 0.044 surface; (**d**) 0.011, (**e**) 0.028, (**f**) 0.044 cross-section.

**Figure 9:**
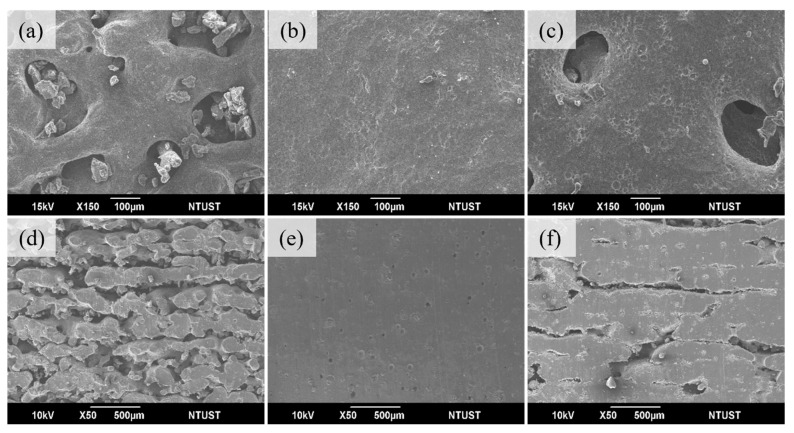
Preheat temperature 75 °C and laser energy (J/mm^2^) used (**a**) 0.011 (**b**) 0.028 surface; (**d**) 0.011 (**e**) 0.028 cross-section and 95 °C laser energy used (**c**) 0.011 (**f**) 0.011 surface and cross-section.

To assess the overall print quality, the laser energy was varied in three steps: 0.011 J/mm^2^, 0.028 J/mm^2^, and 0.044 J/mm^2^. Figure 7, Figure 8 and Figure 9 show the effect of energy variation at three preheat temperatures of 35 °C, 55 °C, and 75 °C, respectively. For a preheated temperature of 35 °C, Figure 7a shows the print surface’s curvature features, which form sintered necks and contain many pores at 0.011 J/mm^2^. Regarding interlayer bonding, individual layers maintain the shape of powder grains, with minimal bonding between layers and more significant gaps (shown in cross-section in Figure 7d), resulting in tensile strength of only 0.1 MPa, elongation at break of 40.7%, and hardness of 24A. Figure 7b shows that the sintered necks on the surface have visibly grown, leaving only a few deeper holes at the energy of 0.028 J/mm^2^. Interlayer bonding shows that individual layers no longer retain the shape of powder grains, and bonding has started to occur. Layers are beginning to bond together, with a trend of reduced gaps (shown in cross-section in Figure 7e). Tensile strength has increased from 0.1 to 1.8 MPa, elongation at break from 40.7% to 137.3%, and hardness from 24A to 65A. Figure 7c shows that the printed surface no longer exhibits noticeable holes, but complete bonding has not occurred, showing a surface with slight irregularities at 0.044 J/mm^2^ energy. There is evident sintering between layers, but some pores are still present (shown in cross-section in Figure 7f). Tensile strength has increased from 1.8 to 3.2 MPa, elongation at break from 137.3% to 164.9%, and hardness from 65A to 72A Table 4.

For a preheat temperature of 55 °C, Figure 8a shows that the printed surface has started to sinter into clusters, but pores remain at 0.011 J/mm^2^ energy. Figure 8d shows the cross-section of interlayer bonding; individual layers no longer retain the appearance of powder grains, and there is a slight tendency for layers to bond together. The tensile strength is 0.4 MPa, elongation at break is 64.5%, and hardness is 41A. Figure 8b shows that the sintered powder clusters on the surface have significantly increased, with only a few pores remaining at the energy of 0.028 J/mm^2^. Interlayer bonding shows multi-layer sintering between layers, but small gaps still exist (cross-section can be observed in Figure 8e). Tensile strength has increased from 0.4 to 3.1 MPa, elongation at break from 64.5% to 182.4%, and hardness from 41A to 70A. Figure 8c shows that the surface no longer exhibits noticeable pores, but complete bonding has not occurred, showing a surface with slight irregularities at 0.044 J/mm^2^ energy. No obvious interlayer gaps exist, with only minor pores remaining (cross-section can be observed in Figure 8f). Tensile strength has increased from 3.1 to 5.6 MPa, elongation at break from 182.4% to 271.8%, and hardness from 70A to 74A, Table 5.

At a preheated temperature of 75 °C, Figure 9a shows that the printed surface has formed large clusters of sintering, but some pores are still present at 0.011 J/mm^2^. Regarding interlayer bonding, individual layers no longer retain the appearance of powder grains, and bonding has begun to occur (cross-section can be observed in Figure 9d). The tensile strength is 1.1 MPa, elongation at break is 104.7%, and hardness is 53A. Figure 9b shows that at the energy of 0.028 J/mm^2^, the surface exhibits large, smooth areas with no visible pores, and there are no obvious interlayer gaps, only tiny micropores remaining. It indicates that the powder has better flowability, promoting fusion between powder particles (cross-section can be observed in Figure 9e). Tensile strength has increased from 1.1 to 7.9 MPa, elongation at break from 104.7% to 364.9%, and hardness from 53A to 78A, Table 6. In Figure 9c, large clusters of sintering appear on the surface, but the powder cannot achieve good melting, resulting in pores remaining on the surface at 0.044 J/mm^2^. Multi-layer sintering between layers can be observed, but the energy cannot fully penetrate the next layer, causing small gaps between layers, as shown in the cross-section in Figure 9f.

With a fixed energy density of 0.028 J/mm^2^, the sintering conditions were observed at different printing temperatures (Figure 9b,e). As the temperature increased from 35 °C to 75 °C, surface sintering evolved from significant growth of sintered necks with only a few deep holes to large, smooth surfaces without any pores—interlayer bonding transitioned from the initial appearance of multi-layer sintering to no noticeable interlayer gaps. Therefore, the hardness increased from 65A, tensile strength from 1.8 MPa, and elongation at break from 137.7% to 78A, 7.9 MPa, and 364.9%, respectively. Hence, it can be inferred that the printing parameters exhibit optimal mechanical strength.

### 3.5. Influence of Carbon Black Added in TPU

Carbon black 0.2, 0.4, and 0.6 wt% were added into TPU to investigate the print quality (TPU02C, TPU04C, and TPU06C). Table 7 shows that TPU02C is the least optimum among the three due to its higher reflectivity than TPU04C and TPU06C, with a value of 17.73%. With temperature and energy density adjustments, the tensile strength increased from 0.3 MPa to 6.1 MPa, elongation at break from 58.6% to 320.7%, hardness from 30A to 74A, and density from 0.48 to 1.01 g/cm^3^. The physical testing results of TPU04C show that with adjustments in temperature and energy density, the tensile strength increased from 0.1 MPa to 7.9 MPa, elongation at break from 40.7% to 364.9%, hardness from 24A to 78A, and density from 0.43 to 1.09 g/cm^3^. The physical testing results of TPU06C show that with adjustments in temperature and energy density, the tensile strength increased from 0.1 MPa to 7.1 MPa, elongation at break from 39.4% to 341.6%, hardness from 24A to 78A, and density from 0.43 to 1.08 g/cm^3^. TPU02C, TPU04C, and TPU06C show that the surface sintering transitioned from a rough appearance to a smooth sintered surface, and the interlayer bonding transitioned from some pores to no obvious interlayer gaps. Consequently, density and hardness showed significant improvements. Regarding tensile strength, when reflectivity decreased from 17.73% to 13.81%, it increased from 6.1 MPa to 7.9 MPa, and elongation at the break increased from 320.7% to 364.9%. However, when reflectivity decreased to 13.34%, tensile strength decreased to 7.1 MPa, and elongation at the break decreased to 341.6%. The printed surface carbon distribution shows that although TPU06C and TPU04C have similar reflectivity, TPU06C has more carbon black adhering to its surface, reducing the contact area during melting compared to TPU04C, leading to a decrease in tensile strength. Hence, it can be inferred that TPU04C, with its reflectivity and carbon black addition amount, exhibits optimal density, elongation rate, hardness, and tensile strength.

**Figure 10:**
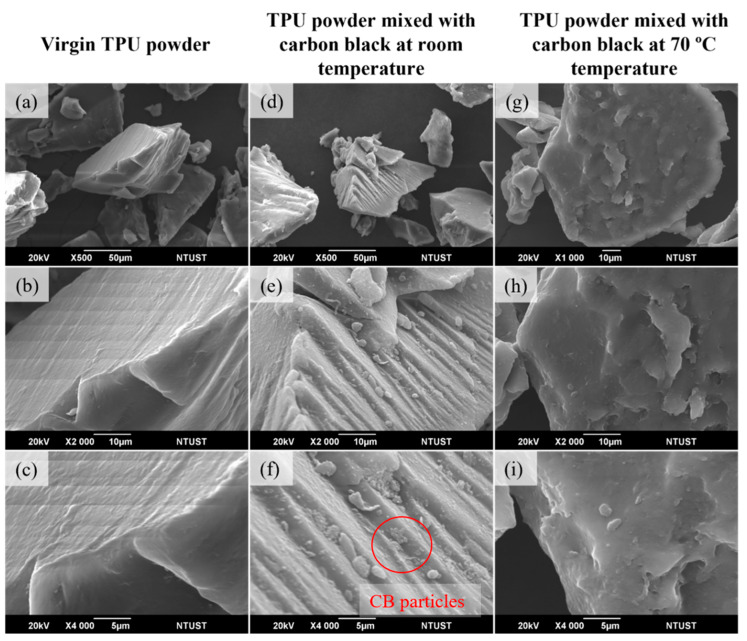
Powder morphology of (**a**–**c**) virgin TPU, (**d**–**f**) TPU powder mixed with carbon black at room temperature, and (**g**–**i**) TPU powder mixed with carbon black at 70 °C temperature.

The SEM images in Figure 10 show the powder morphology of (a–c) virgin TPU, (d–f) TPU powder mixed with carbon black at room temperature, and (g–i) TPU powder mixed with carbon black at 70 °C temperature. The virgin TPU powder was characterized by sharp edges and striations all over the particle surface. Figure 10a–c show the virgin powder has a sharp shape and a clean surface. It could be seen from Figure 10d–f that the appearance of TPU powder mixed with CB at room temperature was still sharp, with finer particles appearing on the surface. This showed that the coating had formed on the surface, changing its surface features. Also, at 70 °C, the temperature enhanced the bonding between the carbon black and TPU polymer, resulting in a uniform coating, unlike the room temperature mixing.

### 3.6. Porosities Observation and Printed Application

**Figure 11:**
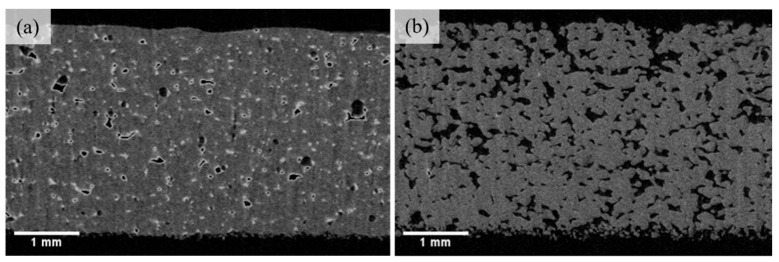
Micro CT-scan of porosity distribution of (**a**) TPU04C and (**b**) Sinterit TPU.

**Figure 12:**
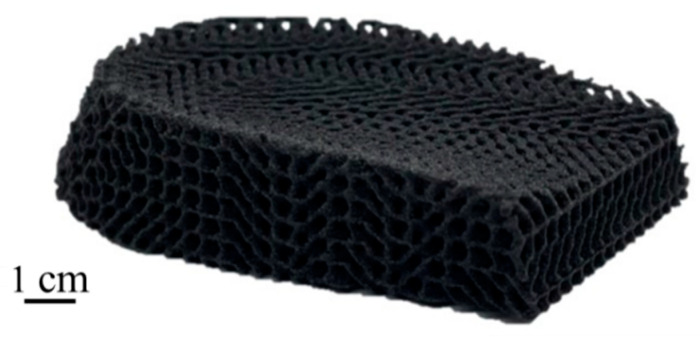
3D printed lattice structure.

The as-printed specimens were conducted using a Bruker Skyscan 1276 (Bruker, Ithaca, NY, USA) micro-computed tomography scanner to observe the porosities. The powder source of TPU we use is different from Sinterit TPU; therefore, a simple comparison was made. TPU04C was printed with a preheating temperature of 75 °C and energy set at 0.028 J/mm^2^, while Sinterit TPU was printed with the manufacturer’s default parameters. Table 8 shows that TPU04C has the highest tensile strength of 7.9 MPa, while Sinterit TPU with 6.3 MPa. Figure 11 shows that the Sinterit TPU exhibits more distributed pores than TPU04C, which only has minimal tiny pores. It can be inferred that powder coated with carbon black with the heating method can lead to better bonding conditions during sintering. Figure 12 shows the printed lattice structures using the TPU04C powder. The formed objects could effectively remove residual powder from the pores. The finished appearance of the objects was intact and exhibited good powder cohesion, allowing them to successfully demonstrate the characteristics of elastic materials when subjected to external pressure.

## 4. Conclusions

The carbon black coating can be uniformly blended with TPU powder without segregation. When carbon black and TPU powder are mixed, reflectance can effectively decrease. As reflectance decreases, pore size and interlayer spacing also decrease. In cases where the laser energy density is insufficient to penetrate the powder layer, the bond between layers within the specimen weakens. Similarly, within the same particle size range, when the layer thickness increases to 150 μm, the laser energy cannot fully penetrate the preceding layer, resulting in pore formation at the layer interface and consequently reducing tensile strength. Incorporating 0.4 wt % carbon black into the TPU powder, a preheating temperature of 75 °C, and a laser energy density of 0.028 J/mm^2^ yields a reflectance of 13.81%. In this study, the powder layer thickness can be increased to 125 μm, resulting in the highest average molding density of 1.09 g/cm^3^, with a tensile strength of 7.9 MPa, elongation at break of 364.9%, and a hardness of 78 A. Excessive carbon black content can decrease the surface area available for powder bonding, thereby impacting tensile strength and elongation rate. Moreover, controlling the energy density and preheating temperature can contribute to achieving a final product with more robust material properties.

## Figures and Tables

**Table 1:** Experimental data on the relationship between carbon black ratio and reflectivity.

Carbon Ratios (%)	0	0.03	0.04	0.05	0.1	0.15	0.2	0.25
Reflectivity (%)	80.12 ±1.69	38.16 ±1.15	34.56 ±0.76	31.00 ±0.82	26.16 ±0.75	22.93 ±0.46	20.67 ±0.58	19.14 ±0.36
Carbon ratios (%)	0.3	0.4	0.5	0.6	0.7	0.8		100
Reflectivity (%)	18.02 ±0.29	15.59 ±0.26	14.47 ±0.34	13.79 ±0.21	13.45 ±0.16	13.06 ±0.18		7.53 ±0.02

Under heating and mixing at 70 °C.

**Table 2:** Printed specimens of I, L, and K dimension error.

No.	Theoretical Thickness	Measured Thickness	Error (%)
I	3.2	4.67	+45.9
K		5.32	+66.3
L		6.84	+113.8

**Table 3:** Layer thickness parameters.

No.	Layer Thickness (μm)	Density (g/cm^3^)	Tensile Strength (MPa)
1	100	1.05	4.1 ± 0.58
2	125	1.09	7.9 ± 0.27
3	150	1.07	5.7 ± 0.43

**Table 4:** TPU04C printed properties @35 °C.

Material Type	Preheat Temp. (°C)	Energy Density (J/mm^2^)	Hardness (Shore A)	Tensile Strength (MPa)	Elongation at Break (%)
		0.011	24	0.1 ± 0.04	40.7 ± 3.6
TPU04C	35	0.028	65	1.8 ± 0.27	137.3 ± 5.3
		0.044	72	3.2 ± 0.29	164.9 ± 7.6

**Table 5:** TPU04C printed properties @55 °C.

Material Type	Preheat Temp. (°C)	Energy Density (J/mm^2^)	Hardness (Shore A)	Tensile Strength (MPa)	Elongation at Break (%)
		0.011	41	0.4 ± 0.05	64.5 ± 4.7
TPU04C	55	0.028	70	3.1± 0.13	182.4 ± 8.4
		0.044	74	5.6 ± 0.42	271.8 ± 6.7

**Table 6:** TPU04C printed properties @75 °C.

Material Type	Preheat Temp. (°C)	Energy Density (J/mm^2^)	Hardness (Shore A)	Tensile Strength (MPa)	Elongation at Break (%)
		0.011	53	1.1 ± 0.05	104.7 ±5.1
TPU04C	75	0.028	78	7.9± 0.27	364.9 ± 6.6
		0.044	NA	NA	NA

**Table 7:** Influence of carbon black in TPU property performances.

Material Type	Density (g/cm^3^)	Hardness (Shore A)	Tensile Strength (MPa)	Elongation at Break (%)	Reflectivity (%)
Virgin TPU	1.11	80	10	>300	80.12 ±1.69
TPU02C	1.01	74	6.1 ± 0.36	320.7 ± 13.6	17.73 ± 0.28
TPU04C	1.09	78	7.9 ± 0.27	364.9 ± 6.6	13.81 ± 0.23
TPU06C	1.08	78	7.1 ± 0.29	341.6 ± 8.3	13.34 ± 0.19

**Table 8:** Mechanical performances of self-mixed TPU powder compared with commercial TPU powder.

	TPU04C	Sinterit TPU
Tensile strength (MPa)	7.9 ± 0.27	6.3 ± 0.56

## Data Availability

The raw data supporting the conclusions of this article will be made available by the authors on request.
